# The German version of the KOOS-Child questionnaire (Knee injury and Osteoarthritis Outcome Score for children) shows a good to excellent internal consistency and a high test–retest reliability in children with knee problems

**DOI:** 10.1007/s00167-022-07074-4

**Published:** 2022-07-30

**Authors:** Cornelia Neuhaus, Carlo Camathias, Marcus Mumme, Oliver Faude

**Affiliations:** 1 Department of Therapy, University Children’s Hospital (UKBB), University of Basel, Spitalstrasse 33, 4056 Basel, Switzerland; 2 Praxis Zeppelin, St. Gallen, Switzerland; 3 Department of Orthopaedics, University Children’s Hospital (UKBB), University of Basel, Basel, Switzerland; 4 Department of Sport, Exercise and Health, Medical Faculty, University of Basel, Basel, Switzerland; 5 Medical Faculty, University of Basel, Basel, Switzerland

**Keywords:** Questionnaire, Youth, Knee injuries, Quality of life, Physical activity, Therapy

## Abstract

**Purpose:**

The Knee Injury Osteoarthritis Outcome Score for children (KOOS-Child) is a self-administered, valid and reliable questionnaire for children and adolescents with knee disorders such as Osgood Schlatter disease, anterior knee pain, and patella dislocation. This study aimed to cross-culturally adapt the German version of the KOOS-Child questionnaire and test the reliability in two groups of children, one treated conservatively and the other surgically.

**Methods:**

A forward–backward translation of the original questionnaire into the German language was conducted. Children and adolescents between 10 and 18 years of age with knee disorders were included. Two groups were compared: sample one consisted of 24 participants with knee pain [20.8% boys; mean age = 13.4 (1.8) years treated conservatively. These participants completed the KOOS-Child questionnaire twice within two weeks to assess test–retest reliability. The second sample included 23 subjects (21.7% boys; mean age = 15.3 (1.9) years] treated surgically due to a knee disorder. They completed the questionnaire before surgery and six months postoperatively. Test–retest reliability and internal consistency were assessed using Spearman’s rank correlation and Cronbach’s alpha.

**Results:**

All subscales showed a good to excellent internal consistency at both measurement points in both groups (conservatively treated group: *a* = 0.88–0.95; surgery group *a* = 0.80–0.91), with the exception of the subscale knee problems (conservatively treated: *a* = 0.60 and 0.52; surgery: *α* = 0.77 and 0.66). Test–retest reliability was between *r* = 0.85 and 0.94.

**Conclusion:**

The predominantly good to excellent internal consistency and the high test–retest reliability justifies the use of the German adaptation of the KOOS-Child questionnaire as a reliable multidimensional instrument for measuring health status and therapeutic effects in adolescents’ knee disorders.

**Supplementary Information:**

The online version contains supplementary material available at 10.1007/s00167-022-07074-4.

## Introduction

Knee pain is widespread in adolescence [6]. To measure the perceived needs of patients with any knee disorder, e.g., regarding symptoms, response to treatment, impact on function, and what is important to them and their families, a Patient-Reported Outcome Measure (PROM) is used. Combined with evidence-based knowledge, PROMs help to better-target medical care to the needs of the patient with the patient actively involved in the treatment process [8].

The KOOS-Child questionnaire is a knee-specific instrument developed to assess patients’ opinions about their knee and associated problems which limit their daily activities and the quality of life [11]. It also evaluates short-term and long-term consequences of knee injury with implications for treatment [14]. The KOOS-Child has been translated into Danish, Finnish, French, Greek, Norwegian, Russian, and Spanish, and validated with excellent to acceptable internal consistency and reliability [4, 9–11, 13, 15]. Since 2018, the International Olympic Committee Consensus Statement on Prevention, Diagnosis, and Management of Pediatric Anterior Cruciate Ligament Injuries group has recommended the KOOS-Child questionnaire as a PROM [1, 13] to assess self-reported knee function.

In the German-speaking area, few standardised questionnaires focusing on knee pain in paediatric orthopaedics exist. This study aimed to conduct a forward–backward translation into German and test the KOOS-Child in a pilot study on children and adolescents with knee problems aged 10–18. We hypothesised that the KOOS-Child questionnaire is highly reliable and consistent in the German language. In the present study, two groups of patients with knee disorders, such as Osgood Schlatter disease, anterior knee pain, and patella dislocation were investigated. The first group was conservatively treated and completed the questionnaire twice within two weeks to measure reliability. The second group received surgery, completing the questionnaire first before surgery and again six months after, to observe treatment progress.

## Materials and methods

Permission to translate the original version from English to German was granted from the original authors. The study protocol complied with the ethical standards of the Declaration of Helsinki and was approved by the local ethics committee (*Ethikkommission Nordwest- und Zentralschweiz, EKNZ, Basel, Switzerland*. Approval Number: 2018-01935). All participants and caregivers gave their written informed consent prior to the start of the study.

### Study design

To assess the test–retest reliability patients completed the questionnaire at two time points approximately two weeks apart either immediately after physiotherapy treatment or soon after, at home. A longitudinal study design (two measurement points before and six months after surgery) was used for patients who underwent surgery.

### Translation and cross-cultural adaptation

The translation consisted of a five-step process [2]. Initially, two qualified, independent German translators translated the English KOOS-Child questionnaire (version LK 2.1) into German. These two physiotherapists, whose mother tongue is German, are proficient in English due to extended overseas residencies. The two independently translated versions of the questionnaire were merged. An expert panel including three physiotherapists obtained a consensus on a preliminary German version. The third part of the process was backward translation of the synthesised German version. Two translators, both native English speakers, retranslated the questionnaire; none had prior knowledge of the original version. All versions of the translated questionnaire (forward and backward translations, synthesised versions) were reviewed. The panel compared the translations and backward translations to obtain a final German version. The original developers of the KOOS questionnaire were involved and informed of these processes.

### Study population

The attending physician or a physiotherapist recruited the participants in one outpatient clinic from January 2019 to June 2019 (conservatively treated group) and February 2020 to May 2020 (patients who underwent surgery). Inclusion criteria were: age between 10 and 18 years, knee pain, speaking sufficient German, being capable of following instructions, and expressing pain. Patients who had undergone surgery within the last three months or with fractures were excluded from the conservative group, whereas surgery that took place after completion of the first questionnaire was an inclusion criterion for the surgery group.

The conservative group consisted of 24 adolescents aged between 10 and 18 years [mean (M) age = 13.4 (1.8) years, 5 boys (20.8%), 19 girls (79.2%)]. Nine participants suffered from anterior knee pain, five from Osgood–Schlatter disease, five from dislocation of the patella, and two from Jumper’s knee syndrome. One person each suffered from an anterior cruciate ligament (ACL) strain, a medial ligament lesion, and pes anserinus syndrome (Table 1). The average time between completing the two questionnaires was 16.3 days (SD 11.2). In the surgical group, 23 adolescents aged 11–18 years (M = 15.3 (1.9) years) completed the questionnaire. Five boys (21.7%) and 18 girls (78.3%) participated in the study (Table 1). Owing to a knee problem, all received surgery, which was shown in Table 2. The time of completion was once before and six to nine months after knee surgery (M = 7.3 months, range 6.0–9.6 months, SD = 1.1).

**Table 1:** Diagnoses of all participants

Diagnosis	Participants		*N* (%)
	Male	Female	Total
Conservative group			
Anterior knee pain	1	8	9 (37.5)
Osgood–Schlatter disease	2	3	5 (20.8)
Patella dislocation	1	4	5 (20.8)
Jumper’s knee	0	2	2 (8.3)
Sprain of the ACL	0	1	1 (4.2)
Medial ligament lesion	1	0	1 (4.2)
Tendinitis anserine	0	1	1 (4.2)
Subtotal	5	19	24
Surgery group			
ACL-rupture	2	2	4 (17.4)
Genua valga	1	2	3 (13.1)
Meniscal cyst	0	1	1 (4.3)
Meniscal lesion	0	4	4 (17.4)
Osteochondrosis dissecans	0	1	1 (4.3)
Patella dislocation	2	4	6 (26.1)
Patellofemoral instability	0	2	2 (8.7)
Posterolateral insufficiency	0	2	2 (8.7)
Subtotal	5	18	23
Total	10	37	47

**Table 2:** Treatments in the surgery group

Surgery	Participants		*N* (%)
	Male	Female	Total
Femoral trochleoplasty with MPFL^a^ reconstruction	1	3	4 (17.4)
Supracondylar femur osteotomy	0	1	1 (4.3)
Hemiepiphysiodesis	1	2	3 (13.1)
Meniscal suture	0	7	7 (30.5)
ACL^b^ reconstruction	1	3	4 (17.4)
Removal of loose fragments	0	2	2 (8.7)
Refixation of the osteochondral flake	1	0	1 (4.3)
Knee joint arthroscopy (shaving)	1	0	1 (4.3)
Total	5	18	23

^a^MPFL: medial patellofemoral ligament

^b^ACL: anterior cruciate ligament

The participants completed the questionnaire independently, whenever possible, directly in the clinic in a separate room or at home. The research staff did not assist; however, they offered standardised instructions for completion.

### Questionnaire

The KOOS-Child questionnaire (Appendix 1) consists of five subscales with 39 items altogether. All subscales refer to the subject’s condition during the past seven days. The first subscale—“knee problems”—consists of seven items (S1–S7) and has questions about movement, extension, and flexion of the knee joint (Appendix 2; Table A). In the next subscale—“how painful”—the respondents are asked about their pain perception during selected movements. The subscale consists of eight items (P1 to P4, P6a, P6b, P8a, and P9) (Appendix 2; Table B). The third subscale focuses on “difficulty during daily activities”. The respondents complete eleven items (A1 to A3, A5, A7, A10, A12 to A14, A16, and A17) (Appendix 2; Table C). In the fourth subscale, the respondents indicate which difficulties they encounter during sports and playing—“difficulty during sports and playing”. Seven items (SP1 to SP5, SPN6, and SPN7) are answered (Appendix 2; Table D). The fifth and last subscale deals with the question “How has your injury affected your life?” (“knee-related quality of life”) with six items (Q1 to Q4, QN5, and QN6) (Appendix 2; Table E). The numbering and labelling of the individual items (e.g., SPN6) were used from the original English version of the KOOS-Child questionnaire, enabling comparisons with the existing studies. Some subscales (e.g., “difficulties with daily activities”) are not consecutively numbered because the KOOS-Child’s items were reduced as compared to the adult version. In each item, one of five possible answers on a 5-point Likert scale can be selected [11]. Each answer has a value between 0 and 4, with 0 representing “no knee pain/knee problems” and 4 representing “severe knee pain/knee problems”. The higher the value, the more severe the difficulties or complications. The respondents were asked to mark which answer was most applicable to them at the time of completion by drawing a cross in a box. If a mark was placed outside a box, the closest option was defined. If two boxes were marked, the box representing the more serious problems and complications was valid [11, 13].

### Statistical analyses

Statistics were performed using the IBM SPSS 20.0 software. The KOOS-Child scores were calculated according to the user guide for each domain with the following formula: [(100 − mean score of the questions)/4] × 100 [12]. The KOOS-Child total score of all subscales was calculated at the two measurement times T1 and T2 (first and second completion date). Raw scores were converted into a scale ranging from 0 to 100, with 0 representing major knee problems and a score of 100 representing no knee problems. For the item statistics, the mean value (M), the standard deviation (SD), and the range of the raw values were calculated for each subscale item. The range could be defined between 0 (no problems) and 4 (considerable problems). For the scale statistics, the mean (M), the standard deviation (SD), and the range of the raw scores were calculated for each of the five subscales. Therefore, the range could also be set between 0 (no problems) and 4 (considerable problems). We analysed internal consistency and test–retest reliability at the two completion times (T1 and T2). The internal consistency is indicated by means of Cronbach’s alpha. The higher the value, the higher the internal consistency. A value ≥ 0.90 is considered excellent, ≥ 0.80 good/high, ≥ 0.70 acceptable, ≥ 0.60 questionable, ≥ 0.50 poor/low, and < 0.50 unacceptable [3]. Test–retest reliability was calculated by means of the Spearman’s rank test. It was calculated as the correlation of a subscale of measurement time one and measurement time two. Higher values indicate higher correlation and thus the stability. An *r* ≥ 0.10 is interpreted as a low/weak correlation, an *r* ≥ 0.30 as a medium/moderate correlation, and an *r* ≥ 0.50 as a high/strong correlation [5]. Paired *t* test was used to compare pre- and postoperative mean scores. We also calculated the change score from T1 to T2 and from pre- to post-surgery and 95% confidence intervals. An a priori power analysis (G*Power) was conducted to estimate the minimum sample size for the reliability analysis. A strong correlation was assumed (test–retest reliability: *r* > 0.70). Assuming a null hypothesis of *r* < 0.30, an alpha level of 0.05, and a power of 0.80, a minimum of 22 respondents was required.

## Results

### Conservative group

#### KOOS-child score

No extreme values (0 and 100) were achieved in scales “knee problems” and “how painful” (Table 3). Mean values at measurement time T2 were similar to those at measurement time T1. Three participants did not mark a box in subscale SP due to a sports dispensation. Detailed range values can be found in Appendix 3, Table A.

**Table 3:** Statistics for the KOOS-Child Score of the five subscales at T1 and T2

	N	T1 (mean, SD^a^)	T2 (mean, SD^a^)	Change score	Confidence interval	*p*-value
S	24	64.6 (22.5)	67.6 (22.0)	3.0	− 1.4 to 7.3	n.s.
P	24	75.1 (17.1)	76.5 (14.5)	1.5	− 2.6 to 5.6	n.s.
ADL	24	73.4 (23.2)	76.7 (21.2)	3.2	− 1.3 to 7.7	n.s.
SP	21	52.9 (29.4)	58.0 (29.2)	5.1	1.3 to 8.9	n.s.
QoL	24	49.8 (21.7)	55.4 (24.9)	5.7	1.3 to 10.0	n.s.

*S* knee problems, *P* how painful, *ADL* difficulty during daily activities, *SP* difficulty in sports and playing, *QoL* knee-related quality of life

^a^SD = standard deviation

#### Item statistics

The mean values at T1 ranged from 0.4 (item S4 of the “knee problems” scale) to 2.7 (item P1 of the “how painful” scale) (Appendix 3, Table B). At T2, the mean values ranged from 0.4 (item S4 of the “knee problems” scale) to 2.3 (item P1 of the “how painful” scale).

#### Scale statistics

The mean scale values at T1 were between 1.1 (scale ADL) and 2.0 (scale QoL) and the standard deviations between 0.7 (scale S) and 1.2 (scale SP). At T2, the mean values were between 0.9 (scale ADL) and 1.8 (scale QoL) and the standard deviations between 0.6 (scale S) and 1.2 (scale SP). All mean values were higher at T1 than at T2 (Appendix 3, Table C).

#### Internal consistency

The internal consistency (Cronbach’s alpha) at T1 was excellent for subscales ADL (*α* = 0.94) and SP (*α* = 0.92), and good for subscales P (*α* = 0.88) and QoL (*α* = 0.88). Only the subscale S achieved a questionable value of *α* = 0.60. At T2, excellent internal consistencies were also found for subscales ADL (*α* = 0.95) and SP (*α* = 0.93) as well as for subscale P (*α* = 0.90). Good internal consistency was found for the subscale QoL (*α* = 0.88). Only the subscale S (*α* = 0.52) showed a poor consistency (Table 4).

**Table 4:** Internal consistency (Cronbach’s alpha) for the five subscales at T1 and T2

Subscale	T1		T2	
	*N*	*α*	*N*	*α*
S	22	0.60	24	0.52
P	22	0.88	22	0.90
ADL	23	0.94	21	0.95
SP	21	0.92	20	0.93
QoL	24	0.88	23	0.88

*S* knee problems, *P* how painful, *ADL* difficulty during daily activities, *SP* difficulty in sports and playing, *QoL* knee-related quality of life

#### Test–retest reliability

Test–retest reliabilities for all five scales were high (Table 5). In the case of the scale function in sport and play, it was only possible to calculate with 21 of 24 test persons since three could not complete the subscale at both time points due to a sports dispensation.

**Table 5:** Test–retest reliability (Spearman correlation) for the five subscales

Subscale	*N*	*r*	95% CI
Knee problems (S)	24	0.85	[0.68; 0.93]
How painful (P)	24	0.87	[0.72; 0.94]
Difficulty during daily activities (ADL)	24	0.86	[0.70; 0.94]
Difficulty in sports and playing (SP)	21	0.94	[0.86; 0.98]
Knee-related quality of life (QoL)	24	0.87	[0.72; 0.94]

For all subscales *p* < 0.001

### Surgery group

#### KOOS-child score

As shown in Table 6 for each subscale, the KOOS-Child scores were calculated from both time points (pre- and post-op). Six months after surgery, the mean values of the KOOS-Child score increased in all scales. Detailed range values can be found in Appendix 4, Table A.

**Table 6:** Statistics for the KOOS-Child Score of the five subscales pre-and post-op

*N* = 23	Pre-op (mean, SD^a^)	Post-op (mean, SD^a^)	Change score	Confidence interval	*p*-value
S	67.6 (20.3)	81.1 (13.4)	13.5	4.0–22.9	< 0.0001
P	51.8 (22.6)	80.9 (15.2)	29.1	18.4–39.8	< 0.0001
ADL	70.5 (20.0)	90.0 (21.6)	19.6	10.5–28.7	< 0.0001
SP	31.8 (24.1)	64.2 (27.3)	32.4	18.1–46.6	< 0.0001
QoL	39.9 (18.4)	66.9 (18.4)	27.0	16.0–38.0	< 0.0001

*S* knee problems, *P* how painful, *ADL* difficulty during daily activities, *SP* difficulty in sports and playing, *QoL* knee-related quality of life

^a^SD = standard deviation

#### Item statistics

Item SP5 had the highest mean value of 3.1 at T1. In general, very high mean values (3.1–2.2) were observed for the subscale SP items. At T2, the mean values of each item were inferior compared with T1 (Appendix 4, Table B).

#### Scale statistics

The descriptive statistics of the subscales present the items of each scale in summary. The mean values at T1 ranged from 1.2 (ADL) to 2.7 (SP). All mean values decreased considerably to 0.4 (ADL) and 1.4 (SP) at the second measurement (Appendix 4, Table C).

#### Internal consistency

According to Cronbach’s alpha, the internal consistencies of the various subscales were in a similar range at the two measurement points. The subscale SP achieved an excellent value, the subscales P, ADL, and QoL a good value for both data sets. Subscale S showed an acceptable internal consistency preoperatively; in the second measurement, the value was in a poor range (Table 7).

**Table 7:** Internal consistency (Cronbach’s alpha) for the five subscales

Subscale	Pre-op		Post-op	
	*N*	*α*	*N*	*α*
S	23	0.77	23	0.66
P	23	0.85	23	0.80
ADL	23	0.89	23	0.88
SP	23	0.90	23	0.91
QoL	23	0.87	23	0.84

*S* knee problems, *P* how painful, *ADL* difficulty during daily activities, *SP* difficulty in sports and playing, *QoL* knee-related quality of life

## Discussion

The most interesting findings of the present study were: first, the KOOS-Child is practical, reliable, and responsive in assessing patient-relevant outcomes in children or adolescents with knee disorders. Second, the KOOS-Child has a good test–retest reliability (*r* > 0.85) for all subscales, and an acceptable internal consistency of *α* > 0.80, except for the subscale knee problems (*α* 0.52–0.77).

The KOOS-Child was translated into the German language and the questionnaire’s psychometric properties evaluated in children with knee disorders. This study found criterion validity was unmeasured since no gold standard was available. The various pathologies affecting our knee pain patients were similar to those found in the previous studies (e.g., ACL rupture, patellar dislocation, anterior knee pain, Osgood–Schlatter disease) [11, 13]. This study’s results for internal consistency are comparable with those in studies from Sweden [11] and Canada [13].

In nearly all subscales, our study presents high internal consistency. The only exception is the subscale “knee problems”, showing questionable or even low internal consistency at both measurement times. However, this subscale also showed lower values in both the Swedish and Canadian studies [11, 13]. For this lower homogeneity, Örtqvist et al. concluded that knee pain in patients is multifaceted [11]. Therefore, since patients have different knee pathologies, items in the subscale “knee problems” can be interpreted differently. Another possible reason for this is, that children have misinterpreted or misread questions S4 (During the past seven days, how often have you been able to fully straighten your knee on your own?) and S5 (During the past seven days, how often have you been able to fully bend your knee on your own?) and answered the opposite way. The response scales of these two questions are inverted (from always to never) compared to the other questions of this subscale (from never to always). The Canadian study also has this assumption and intends to review and retest this in a revised version of the questionnaire [13].

There is a broad age variability (10–18 years) in our study group. However, the age range is similar to previously studied populations (10–16 years in Örtqvist et al. and 8–16 years in Rioux Trottier et al. [11, 13]). The various pathologies presenting in our knee patients were similar to those found in the other two studies. However, in our study, in the conservative group, a higher percentage of youth (37.5%) suffered from anterior knee pain compared to Örtqvist et al. (6%) and Rioux Trottier et al. (14.9%) [11, 13]. In this study, the time difference between T1 and T2 was 16 days (SD = 11.2), compared to 11 days (SD = 4.2) in the survey by Örtqvist et al. [11].

Hill et al. studied a mean recovery profile 3, 6, and 12 months after surgery [7]. This information is valuable for preoperative patients and provides reassurance during the months of rehabilitation. The study cannot be directly compared to the KOOS questionnaire for adults, as only patients with ACL rupture were included. The subscale scores indicate that six months after the operation,patients had decreased symptoms, pain, limitations in daily life, and sports. In addition, quality of life increased post-surgery: mean changes after surgery are similar to those found in our study [7].

Van der Velden et al. compared the Pedi-IKDC with the KOOS-Child questionnaire in children with different knee conditions. The Pedi-IKDC showed good responsiveness, the KOOS-Child showed good responsiveness in two subscales (ADL and QoL), and only moderate responsiveness in the subscales Symptoms, Pain, and Sport/Play. The authors therefore recommend favouring the Pedi-IKDC to the KOOS-Child, as it showed better psychometric properties [16]. However, as far as we know, no German version of the Pedi-IKDC questionnaire is currently available.

A limitation of our study is that the questionnaire was not tested for comprehensibility in the first step by interviewing the participants. However, children and adolescents were able to answer all questions, so comprehension is assumed. For various reasons, the questionnaire could not always be completed under standardised conditions and it is also difficult to say how much influence the parents had, especially for the younger participants. Despite these differences in implementation, the questionnaire proved to be reliable and we assume that the place and time of completion has no influence. A further limitation is the small sample size of *n* = 47, within which there is a high proportion of anterior knee pain patients (37.5%) and unequal sex ratio (10 males, 37 females). The results for the male participants need to be interpreted with caution. According to the Canadian study, gender effects should be considered when evaluating the KOOS-Child subscale score [13]. Owing to the lack of comparative instruments, the questionnaire could also not be tested for validity. To the best of our knowledge, this is the first study to compare the KOOS-Child questionnaire before surgery and six months after. Future studies will help illuminate potential problems with long-term follow-up of children with knee disease. Researchers should investigate the psychometric properties and performance of the KOOS-Child in other groups of children with knee disorders (e.g., only children with patella dislocation).

## Conclusion

The German adaptation of the KOOS-Child questionnaire has a good to excellent internal consistency and high test–retest reliability. It is a reliable multidimensional instrument to measure health status or therapeutic effects in children and adolescents with knee disorders.

## Supplementary Information

Below is the link to the electronic supplementary material.

Supplementary file1 (DOCX 126 KB)
