# Supplementary material for: The German version of the KOOS-Child questionnaire (Knee injury and Osteoarthritis Outcome Score for children) shows a good to excellent internal consistency and a high test–retest reliability in children with knee problems
Source: Knee Surg Sports Traumatol Arthrosc. 2022 Jul 30;31(4):1354–60. doi: 10.1007/s00167-022-07074-4 (PMC10050051; doi:10.1007/s00167-022-07074-4)
Supplement: Supplementary file 1 — Supplementary file1 (DOCX 126 KB) [file 167_2022_7074_MOESM1_ESM.docx]

**Appendix 1: KOOS-Child Questionnaire**

**KOOS-Kind KNIE FRAGEBOGEN**

Heutiges Datum: _____________________ Geburtsdatum: ____________________

Name: ______________________________________________________________

**EINLEITUNG**

Diese Fragen sammeln Informationen darüber, wie dich dein verletztes Knie beeinflusst.

Bitte beantworte jede Frage, indem du das für dich passende Kästchen ankreuzt, pro Frage

nur ein Kreuz. Wenn du unsicher bist, wie du eine Frage beantworten sollst, wähle die für

dich am besten passende Antwort aus.

**KNIEPROBLEME**

| S1. Wie oft war dein Knie in den letzten 7 Tagen geschwollen? | | | | |
| --- | --- | --- | --- | --- |
| nie | selten | manchmal | oft | immer |
| **􀀀** | **􀀀** | **􀀀** | **􀀀** | **􀀀** |
| S2. Wie oft hat dein Knie in den letzten 7 Tagen irgendwelche Geräusche (z.B. ein Knirschen, ein Klicken) gemacht? | | | | |
| nie | selten | manchmal | oft | immer |
| **􀀀** | **􀀀** | **􀀀** | **􀀀** | **􀀀** |
| S3. Wie oft war dein Knie in den letzten 7 Tagen blockiert, wenn du es bewegt hast? | | | | |
| nie | selten | manchmal | oft | immer |
| **􀀀** | **􀀀** | **􀀀** | **􀀀** | **􀀀** |
| S4. Wie oft konntest du dein Knie in den letzten 7 Tagen selbständig ganz strecken? | | | | |
| immer | oft | manchmal | selten | nie |
| **􀀀** | **􀀀** | **􀀀** | **􀀀** | **􀀀** |
| S5. Wie oft konntest du dein Knie in den letzten 7 Tagen selbständig ganz beugen? | | | | |
| immer | oft | manchmal | selten | nie |
| **􀀀** | **􀀀** | **􀀀** | **􀀀** | **􀀀** |
| S6. Wie schwierig war es in den letzten 7 Tagen für dich, direkt nach dem Aufwachen am Morgen dein Knie zu bewegen? | | | | |
| überhaupt nicht schwierig | wenig schwierig | mässig schwierig | schwierig | sehr schwierig |
| **􀀀** | **􀀀** | **􀀀** | **􀀀** | **􀀀** |
| S7. Wie schwierig war es für dich in den letzten 7 Tagen, dein Knie später am Tag zu bewegen, nachdem du eine Weile gesessen bist? | | | | |
| überhaupt nicht schwierig | wenig schwierig | mässig schwierig | schwierig | sehr schwierig |
| **􀀀** | **􀀀** | **􀀀** | **􀀀** | **􀀀** |
| P1. Wie oft hattest du im letzten Monat Knieschmerzen? | | | | |
| nie | selten | manchmal | oft | die ganze Zeit |
| **􀀀** | **􀀀** | **􀀀** | **􀀀** | **􀀀** |

**WIE SCHMERZHAFT**

**Wie starke Knieschmerzen** hattest du **in den letzten 7 Tagen** während den folgenden

Aktivitäten? Kreuze die am meisten passende Antwort für jede Frage an.

|  | keine  Schmerzen | ein wenig  Schmerzen | mässige  Schmerzen | starke Schmerzen | sehr starke Schmerzen |
| --- | --- | --- | --- | --- | --- |
| P2. Bei Drehbewegungen auf deinem verletzten Knie während dem Gehen/Stehen/ Rennen |  |  |  |  |  |
| P3. Beim vollen Strecken des verletzten Knies |  |  |  |  |  |
| P4. Beim vollen Beugen des verletzten Knies |  |  |  |  |  |
| P6a. Beim Treppen Hinaufsteigen |  |  |  |  |  |
| P6b. Beim Treppen Hinuntergehen |  |  |  |  |  |
| P8a. Beim Sitzen mit deinem verletzten Knie in gebeugter Position |  |  |  |  |  |
| P9. Beim Aufrechtstehen auf beiden Beinen für eine gewisse Zeit |  |  |  |  |  |

**SCHWIERIGKEITEN BEI TÄGLICHEN AKTIVITÄTEN**

| A1. Wie schwierig war es in den letzten 7 Tagen für dich, Treppen hinunterzusteigen? | | | | |
| --- | --- | --- | --- | --- |
| überhaupt nicht schwierig | wenig schwierig | mässig schwierig | schwierig | sehr schwierig |
| **􀀀** | **􀀀** | **􀀀** | **􀀀** | **􀀀** |
| A2. Wie schwierig war es in den letzten 7 Tagen für dich, Treppen hinaufzusteigen? | | | | |
| überhaupt nicht schwierig | wenig schwierig | mässig schwierig | schwierig | sehr schwierig |
| **􀀀** | **􀀀** | **􀀀** | **􀀀** | **􀀀** |
| A3. Wie schwierig war es in den letzten 7 Tagen für dich, von einem Stuhl aufzustehen? | | | | |
| überhaupt nicht schwierig | wenig schwierig | mässig schwierig | schwierig | sehr schwierig |
| **􀀀** | **􀀀** | **􀀀** | **􀀀** | **􀀀** |
| A5. Wie schwierig war es für dich in den letzten 7 Tagen, dich zu bücken und etwas vom Boden aufzuheben? | | | | |
| überhaupt nicht schwierig | wenig schwierig | mässig schwierig | schwierig | sehr schwierig |
| **􀀀** | **􀀀** | **􀀀** | **􀀀** | **􀀀** |
| A7. Wie schwierig war es in den letzten 7 Tagen für dich, in ein Auto ein- und auszusteigen? | | | | |
| überhaupt nicht schwierig | wenig schwierig | mässig schwierig | schwierig | sehr schwierig |
| **􀀀** | **􀀀** | **􀀀** | **􀀀** | **􀀀** |

| A10. Wie schwierig war es in den letzten 7 Tagen für dich, aus dem Bett aufzustehen? | | | | |
| --- | --- | --- | --- | --- |
| überhaupt nicht schwierig | wenig schwierig | mässig schwierig | schwierig | sehr schwierig |
| **􀀀** | **􀀀** | **􀀀** | **􀀀** | **􀀀** |
| A12. Wie schwierig war es in den letzten 7 Tagen für dich, deine Knieposition zu verändern, wenn du im Bett lagst? | | | | |
| überhaupt nicht schwierig | wenig schwierig | mässig schwierig | schwierig | sehr schwierig |
| **􀀀** | **􀀀** | **􀀀** | **􀀀** | **􀀀** |
| A13. Wie schwierig war es in den letzten 7 Tagen für dich, in die Badewanne/ Dusche ein- oder auszusteigen? | | | | |
| überhaupt nicht schwierig | wenig schwierig | mässig schwierig | schwierig | sehr schwierig |
| **􀀀** | **􀀀** | **􀀀** | **􀀀** | **􀀀** |
| A14. Wie schwierig war es in den letzten 7 Tagen für dich, mit gebeugtem verletzten Knie auf einen Stuhl zu sitzen? | | | | |
| überhaupt nicht schwierig | wenig schwierig | mässig schwierig | schwierig | sehr schwierig |
| **􀀀** | **􀀀** | **􀀀** | **􀀀** | **􀀀** |
| A16. Wie schwierig war es in den letzten 7 Tagen für dich, schwere Taschen oder einen Rucksack etc. zu tragen? | | | | |
| überhaupt nicht schwierig | wenig schwierig | mässig schwierig | schwierig | sehr schwierig |
| **􀀀** | **􀀀** | **􀀀** | **􀀀** | **􀀀** |
| A17. Wie schwierig war es in den letzten 7 Tagen für dich, kleinere Hausarbeiten zu erledigen wie dein Zimmer aufräumen, die Abwaschmaschine ein- oder ausräumen, dein Bett machen etc.? | | | | |
| überhaupt nicht schwierig | wenig schwierig | mässig schwierig | schwierig | sehr schwierig |
| **􀀀** | **􀀀** | **􀀀** | **􀀀** | **􀀀** |

**SCHWIERIGKEITEN BEI SPORT- UND FREIZEITAKTIVITÄTEN**

| SP1. Wie schwierig war es für dich in den letzten 7 Tagen, dich bei Sport- und Freizeitaktivitäten zu bücken/knien? | | | | | 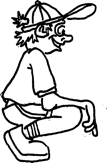 |
| --- | --- | --- | --- | --- | --- |
| überhaupt nicht schwierig | wenig schwierig | mässig schwierig | schwierig | sehr schwierig |  |
| **􀀀** | **􀀀** | **􀀀** | **􀀀** | **􀀀** |  |
| SP2. Wie schwierig war es in den letzten 7 Tagen für dich, während Sport und Freizeitaktivitäten zu rennen? | | | | | 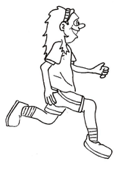 |
| überhaupt nicht schwierig | wenig schwierig | mässig schwierig | schwierig | sehr schwierig |  |
| **􀀀** | **􀀀** | **􀀀** | **􀀀** | **􀀀** |  |
| SP3. Wie schwierig war es in den letzten 7 Tagen für dich, während Sport und Freizeitaktivitäten in die Höhe zu springen? | | | | | 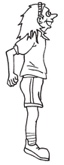 |
| überhaupt nicht schwierig | wenig schwierig | mässig schwierig | schwierig | sehr schwierig |  |
| **􀀀** | **􀀀** | **􀀀** | **􀀀** | **􀀀** |  |
| SP4. Wie schwierig war es in den letzten 7 Tagen für dich aufgrund deines verletzten Knies, dich während Sport und Freizeitaktivitäten zu drehen? | | | | | 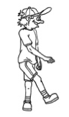 |
| überhaupt nicht schwierig | wenig schwierig | mässig schwierig | schwierig | sehr schwierig |  |
| **􀀀** | **􀀀** | **􀀀** | **􀀀** | **􀀀** |  |

| SP5. Wie viel Schwierigkeiten hattest du aufgrund deines verletzten Knies in den letzten 7 Tagen dabei, zu knien? | | | | | 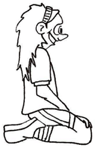 |
| --- | --- | --- | --- | --- | --- |
| überhaupt nicht schwierig | wenig schwierig | mässig schwierig | schwierig | sehr schwierig |  |
| **􀀀** | **􀀀** | **􀀀** | **􀀀** | **􀀀** |  |
| SPN6. Wie viel Schwierigkeiten hattest du in den letzten 7 Tagen dabei, beim Gehen/Rennen auf unebenem Boden dein Gleichgewicht zu behalten? | | | | | 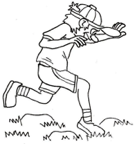 |
| überhaupt nicht schwierig | wenig schwierig | mässig schwierig | schwierig | sehr schwierig |  |
| **􀀀** | **􀀀** | **􀀀** | **􀀀** | **􀀀** |  |
| SPN7. Wie viel Schwierigkeiten hattest du aufgrund deines verletzten Knies in den letzten 7 Tagen dabei, Sport zu treiben? | | | | | 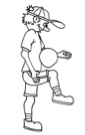 |
| überhaupt nicht schwierig | wenig schwierig | mässig schwierig | schwierig | sehr schwierig |  |
| **􀀀** | **􀀀** | **􀀀** | **􀀀** | **􀀀** |  |

**INWIEFERN IST DEIN LEBEN DURCH DEIN VERLETZTES KNIE**

**BEEINTRÄCHTIGT?**

| Q1. Wie oft denkst Du über deine Knieprobleme nach? | | | | |
| --- | --- | --- | --- | --- |
| nie | wenig | manchmal | oft | die ganze Zeit |
| **􀀀** | **􀀀** | **􀀀** | **􀀀** | **􀀀** |
| Q2. Wie stark hast du deine Lebensgewohnheiten aufgrund der Knieverletzung geändert? | | | | |
| überhaupt nicht | ein wenig | etwas | stark | sehr stark |
| **􀀀** | **􀀀** | **􀀀** | **􀀀** | **􀀀** |
| Q3. Wie sehr vertraust du deinem verletzten Knie? | | | | |
| vollständig | weitgehend | teilweise | kaum | überhaupt nicht |
| **􀀀** | **􀀀** | **􀀀** | **􀀀** | **􀀀** |
| Q4. Wie viele Probleme hast du mit deinem verletzten Knie insgesamt? | | | | |
| keine | wenig | ab und zu | viel | extrem |
| **􀀀** | **􀀀** | **􀀀** | **􀀀** | **􀀀** |
| QN5. Wie schwierig war es für dich aufgrund deines verletzten Knies, in die Schule zu kommen oder in der Schule herum zu gehen (Treppensteigen, Türe öffnen, Bücher tragen, in der Pause mitspielen)? | | | | |
| überhaupt nicht schwierig | wenig schwierig | mässig schwierig | schwierig | sehr schwierig |
| **􀀀** | **􀀀** | **􀀀** | **􀀀** | **􀀀** |
| QN6. Wie schwierig war es für dich aufgrund deines verletzten Knies, mit Freunden etwas zu unternehmen? | | | | |
| überhaupt nicht schwierig | wenig schwierig | mässig schwierig | schwierig | sehr schwierig |
| **􀀀** | **􀀀** | **􀀀** | **􀀀** | **􀀀** |

**Vielen Dank für das Ausfüllen aller Fragen in diesem Fragebogen!**

**Appendix 2: KOOS-Child Subscales**

**Table A**

Subscale: Knee problems (S)

| Abbreviation | Item |
| --- | --- |
| S1 | Wie oft war dein Knie in den letzten 7 Tagen geschwollen? |
| S2 | Wie oft hat dein Knie in den letzten 7 Tagen irgendwelche Geräusche (z. B. ein Knirschen, ein Klicken) gemacht? |
| S3 | Wie oft war dein Knie in den letzten 7 Tagen blockiert, wenn du es bewegt hast? |
| S4 | Wie oft konntest du dein Knie in den letzten 7 Tagen selbständig ganz strecken? |
| S5 | Wie oft konntest du dein Knie in den letzten 7 Tagen selbständig ganz beugen? |
| S6 | Wie schwierig war es in den letzten 7 Tagen für dich, direkt nach dem Aufwachen am Morgen dein Knie zu bewegen? |
| S7 | Wie schwierig war es für dich in den letzten 7 Tagen, dein Knie später am Tag zu bewegen, nachdem du eine Weile gesessen bist? |

**Table B**

Subscale: How painful (P)

| Abbreviation | Item |
| --- | --- |
| P1 | Wie oft hattest du im letzten Monat Knieschmerzen? |
|  | Wie starke Knieschmerzen hattest du in den letzten 7 Tagen während den folgenden Aktivitäten? |
| P2 | Bei Drehbewegungen auf deinem verletzten Knie während dem Gehen / Stehen / Rennen |
| P3 | Beim vollen Strecken des verletzten Knies |
| P4 | Beim vollen Beugen des verletzen Knies |
| P6a | Beim Treppen Hinaufsteigen |
| P6b | Beim Treppen Hinuntergehen |
| P8a | Beim Sitzen mit deinem verletzten Knie in gebeugter Position |
| P9 | Beim Aufrechtstehen auf beiden Beinen für eine gewisse Zeit |

**Table C**

Subscale: Difficulty during daily activities (ADL)

| Abbreviation | Item |
| --- | --- |
| A1 | Wie schwierig war es in den letzten 7 Tagen für dich, Treppen hinunterzusteigen? |
| A2 | Wie schwierig war es in den letzten 7 Tagen für dich, Treppen hinaufzusteigen? |
| A3 | Wie schwierig war es in den letzten 7 Tagen für dich, von einem Stuhl aufzustehen? |
| A5 | Wie schwierig war es für dich in den letzten 7 Tagen, dich zu bücken und etwas vom Boden aufzuheben? |
| A7 | Wie schwierig war es in den letzten 7 Tagen für dich, in ein Auto ein- oder auszusteigen? |
| A10 | Wie schwierig war es in den letzten 7 Tagen für dich, aus dem Bett aufzustehen? |
| A12 | Wie schwierig war es in den letzten 7 Tagen für dich, deine Knieposition zu verändern, wenn du im Bett lagst? |
| A13 | Wie schwierig war es in den letzten 7 Tagen für dich, in die Badewanne / Dusche ein- oder auszusteigen? |
| A14 | Wie schwierig war es in den letzten 7 Tagen für dich, mit gebeugtem verletztem Knie auf einen Stuhl zu sitzen? |
| A16 | Wie schwierig war es in den letzten 7 Tagen für dich, schwere Taschen oder einen Rucksack etc. zu tragen? |
| A17 | Wie schwierig war es in den letzten 7 Tagen für dich, kleinere Hausarbeiten zu erledigen wie dein Zimmer aufräumen, die Abwaschmaschine ein- oder ausräumen, dein Bett machen etc.? |

**Table D**

Subscale: Difficulty during sports and playing (SP)

| Abbreviation | Item |
| --- | --- |
| SP1 | Wie schwierig war es für dich in den letzten 7 Tagen, dich bei Sport- und Freizeitaktivitäten zu bücken/knien? |
| SP2 | Wie schwierig war es in den letzten 7 Tagen für dich, während Sport- und Freizeitaktivitäten zu rennen? |
| SP3 | Wie schwierig war es in den letzten 7 Tagen für dich, während Sport- und Freizeitaktivitäten in die Höhe zu springen? |
| SP4 | Wie schwierig war es in den letzten 7 Tagen für dich aufgrund deines verletzten Knies, dich während Sport- und Freizeitaktivitäten zu drehen? |
| SP5 | Wie viel Schwierigkeiten hattest du aufgrund deines verletzten Knies in den letzten 7 Tagen dabei, zu knien? |
| SPN6 | Wie viel Schwierigkeiten hattest du in den letzten 7 Tagen dabei, beim Gehen/ Rennen auf unebenem Boden dein Gleichgewicht zu behalten? |
| SPN7 | Wie viel Schwierigkeiten hattest du aufgrund deines verletzten Knies in den letzten 7 Tagen dabei, Sport zu treiben? |

**Table E**

Subscale: How has your injury affected your life? (QoL)

| Abbreviation | Item |
| --- | --- |
| Q1 | Wie oft denkst du über deine Knieprobleme nach? |
| Q2 | Wie stark hast du deine Lebensgewohnheiten aufgrund der Knieverletzung geändert? |
| Q3 | Wie sehr vertraust du deinem verletzten Knie? |
| Q4 | Wie viele Probleme hast du mit deinem verletzten Knie insgesamt? |
| QN5 | Wie schwierig war es für dich aufgrund deines verletzten Knies, in die Schule zu kommen oder in der Schule herum zu gehen (Treppensteigen, Türe öffnen, Bücher tragen, in der Pause mitspielen)? |
| QN6 | Wie schwierig war es für dich aufgrund deines verletzten Knies, mit Freunden etwas zu unternehmen? |

**Appendix 3 Conservative Group**

**Table A. Descriptive statistics for the KOOS-Child score of the five subscales at T1 and T2**

| Subscale | T1 | | | | T2 | | | |
| --- | --- | --- | --- | --- | --- | --- | --- | --- |
|  | N | M | SD | Range | N | M | SD | Range |
| S | 24 | 64.62 | 22.51 | 16–97 | 24 | 67.63 | 21.99 | 25–97 |
| P | 24 | 75.05 | 17.12 | 43– 96 | 24 | 76.49 | 14.48 | 50–100 |
| ADL | 24 | 73.40 | 23.19 | 23–100 | 24 | 76.65 | 21.19 | 36–100 |
| SP | 21 | 52.89 | 29.38 | 0–100 | 21 | 58.02 | 29.19 | 4–100 |
| QoL | 24 | 49.83 | 21.65 | 0–79 | 24 | 55.38 | 24.92 | 8–88 |

S = knee problems; P = how painful; ADL = difficulty during daily activities; SP = difficulty in sports and playing; QoL = knee-related quality of life

**Table B. Descriptive statistics for the KOOS-Child Score of the five subscales at T1 and T2 (Item statistics)**

| Items | T1 | | | | T2 | | | |
| --- | --- | --- | --- | --- | --- | --- | --- | --- |
|  | *N* | *M* | *SD* | Range | *N* | *M* | *SD* | Range |
| Knee Problems (S) |  |  |  |  |  |  |  |  |
| S1 | 24 | 1.37 | 1.69 | 0 – 4 | 24 | 0.96 | 1.40 | 0 – 4 |
| S2 | 24 | 1.54 | 1.38 | 0 – 4 | 24 | 1.46 | 1.23 | 0 – 4 |
| S3 | 23 | 0.96 | 1.02 | 0 – 3 | 24 | 0.92 | 1.06 | 0 – 3 |
| S4 | 24 | 0.42 | 0.83 | 0 – 3 | 24 | 0.42 | 0.78 | 0 – 2 |
| S5 | 23 | 0.74 | 1.29 | 0 – 4 | 24 | 0.87 | 1.36 | 0 – 4 |
| S6 | 24 | 1.21 | 1.10 | 0 – 3 | 24 | 0.96 | 1.08 | 0 – 4 |
| S7 | 24 | 1.29 | 1.12 | 0 – 3 | 24 | 1.17 | 1.00 | 0 – 3 |
| How painful (P) |  |  |  |  |  |  |  |  |
| P1 | 23 | 2.65 | 1.07 | 0 – 4 | 24 | 2.33 | 1.01 | 1 – 4 |
| P2 | 23 | 1.61 | 1.31 | 0 – 4 | 24 | 1.75 | 1.29 | 0 – 4 |
| P3 | 24 | 1.08 | 1.06 | 0 – 4 | 24 | 0.67 | 0.82 | 0 – 3 |
| P4 | 24 | 1.29 | 1.37 | 0 – 4 | 24 | 1.00 | 1.18 | 0 – 4 |
| P6a | 24 | 1.50 | 1.38 | 0 – 4 | 24 | 1.46 | 1.38 | 0 – 4 |
| P6b | 24 | 0.79 | 1.02 | 0 – 3 | 23 | 0.83 | 1.15 | 0 – 3 |
| P8a | 24 | 1.08 | 1.35 | 0 – 4 | 24 | 0.92 | 1.28 | 0 – 4 |
| P9 | 23 | 1.39 | 1.41 | 0 – 4 | 23 | 1.26 | 1.32 | 0 – 4 |
| Difficulty during daily  activities (ADL) |  |  |  |  |  |  |  |  |
| A1 | 24 | 1.04 | 1.04 | 0 – 4 | 24 | 0.96 | 1.23 | 0 – 4 |
| A2 | 24 | 1.46 | 1.32 | 0 – 4 | 24 | 1.38 | 1.41 | 0 – 4 |
| A3 | 23 | 0.87 | 1.01 | 0 – 3 | 24 | 0.83 | 0.87 | 0 – 3 |
| A5 | 24 | 1.54 | 1.56 | 0 – 4 | 24 | 1.54 | 1.41 | 0 – 4 |
| A7 | 24 | 1.04 | 1.08 | 0 – 4 | 23 | 0.57 | 0.79 | 0 – 3 |
| A10 | 24 | 0.88 | 0.90 | 0 – 3 | 24 | 0.83 | 0.92 | 0 – 3 |
| A12 | 24 | 0.79 | 0.83 | 0 – 3 | 23 | 0.61 | 0.78 | 0 – 2 |
| A13 | 24 | 1.04 | 1.20 | 0 – 4 | 24 | 0.83 | 1.09 | 0 – 3 |
| A14 | 24 | 1.13 | 1.23 | 0 – 3 | 23 | 0.65 | 0.94 | 0 – 3 |
| A16 | 24 | 1.17 | 1.44 | 0 – 4 | 24 | 0.96 | 1.08 | 0 – 4 |
| A17 | 24 | 0.92 | 1.06 | 0 – 3 | 24 | 0.92 | 1.10 | 0 – 3 |
| Difficulty in sports and  playing (SP) |  |  |  |  |  |  |  |  |
| SP1 | 23 | 2.26 | 1.36 | 0 – 4 | 21 | 2.05 | 1.50 | 0 – 4 |
| SP2 | 21 | 1.81 | 1.47 | 0 – 4 | 21 | 1.67 | 1.43 | 0 – 4 |
| SP3 | 21 | 1.81 | 1.60 | 0 – 4 | 21 | 1.52 | 1.37 | 0 – 4 |
| SP4 | 21 | 1.52 | 1.54 | 0 – 4 | 21 | 1.24 | 1.41 | 0 – 4 |
| SP5 | 22 | 2.23 | 1.51 | 0 – 4 | 22 | 1.95 | 1.36 | 0 – 4 |
| SPN6 | 23 | 1.52 | 1.31 | 0 – 4 | 22 | 1.45 | 1.41 | 0 – 4 |
| SPN7 | 21 | 2.10 | 1.34 | 0 – 4 | 20 | 1.90 | 1.37 | 0 – 4 |
| Knee-related  quality of life (QoL) |  |  |  |  |  |  |  |  |
| Q1 | 24 | 2.42 | 0.88 | 1 – 4 | 24 | 2.08 | 1.10 | 0 – 4 |
| Q2 | 24 | 1.67 | 1.17 | 0 – 4 | 24 | 1.71 | 1.20 | 0 – 4 |
| Q3 | 24 | 1.96 | 1.00 | 0 – 4 | 24 | 1.83 | 1.09 | 0 – 4 |
| Q4 | 24 | 2.42 | 0.88 | 1 – 4 | 24 | 2.25 | 1.03 | 0 – 4 |
| QN5 | 24 | 2.00 | 1.41 | 0 – 4 | 23 | 1.30 | 1.52 | 0 – 4 |
| QN6 | 24 | 1.25 | 1.45 | 0 – 4 | 23 | 1.17 | 1.27 | 0 – 4 |

**Table C. Descriptive statistics for the KOOS-Child Score of the five subscales at T1 and T2 (Scale statistics)**

| Subscale | T1 | | | | T2 | | | |
| --- | --- | --- | --- | --- | --- | --- | --- | --- |
|  | *N* | *M* | *SD* | Range | *N* | *M* | *SD* | Range |
| S | 24 | 1.09 | 0.69 | 0.14 – 2.29 | 24 | 0.96 | 0.59 | 0.00 – 2.00 |
| P | 24 | 1.42 | 0.89 | 0.13 – 3.38 | 24 | 1.29 | 0.89 | 0.13 – 3.00 |
| ADL | 24 | 1.08 | 0.91 | 0.00 – 3.09 | 24 | 0.92 | 0.85 | 0.00 – 2.55 |
| SP | 23 | 1.91 | 1.21 | 0.00 – 4.00 | 23 | 1.70 | 1.16 | 0.00 – 3.86 |
| QoL | 24 | 1.95 | 0.91 | 0.67 – 4.00 | 24 | 1.76 | 1.00 | 0.50 – 3.67 |

S = knee problems; P = how painful; ADL = difficulty during daily activities; SP = difficulty in sports and playing; QoL = knee-related quality of life

**Appendix 4 Surgery group**

**Table A. Descriptive statistics for the KOOS-Child Score of the five subscales at T1 and T2**

| Subscale | T1 | | | | T2 | | | |
| --- | --- | --- | --- | --- | --- | --- | --- | --- |
|  | N | M | SD | Range | N | M | SD | Range |
| S | 23 | 67.61 | 20.29 | 14 – 94 | 23 | 81.06 | 13.43 | 50 – 100 |
| P | 23 | 51.78 | 22.57 | 12 – 97 | 23 | 80.85 | 15.20 | 53 – 100 |
| ADL | 23 | 70.45 | 20.00 | 22 – 97 | 23 | 90.02 | 21.55 | 52 – 100 |
| SP | 23 | 31.83 | 24.12 | 0 – 92 | 23 | 64.21 | 27.25 | 7 – 100 |
| QoL | 23 | 39.86 | 18.37 | 8 – 75 | 23 | 66.85 | 18.44 | 16 – 100 |

S = knee problems; P = how painful; ADL = difficulty during daily activities; SP = difficulty in sports and playing; QoL = knee-related quality of life

**Table B. Descriptive statistics for the KOOS-Child Score of the five subscales at T1 and T2 (Item statistics)**

| Item | T1 | | | | T2 | | | |
| --- | --- | --- | --- | --- | --- | --- | --- | --- |
|  | *N* | *M* | *SD* | Range | *N* | *M* | *SD* | Range |
| Knee Problems (S) |  |  |  |  |  |  |  |  |
| S1 | 23 | 1.17 | 1.37 | 0 – 4 | 23 | 0.35 | 0.57 | 0 – 2 |
| S2 | 23 | 2.13 | 1.10 | 0 – 4 | 23 | 1.96 | 1.26 | 0 – 4 |
| S3 | 23 | 1.00 | 1.00 | 0 – 4 | 23 | 0.78 | 0.90 | 0 – 3 |
| S4 | 23 | 0.74 | 1.29 | 0 – 4 | 23 | 0.30 | 0.77 | 0 – 3 |
| S5 | 23 | 1.13 | 1.39 | 0 – 4 | 23 | 0.52 | 0.95 | 0 – 3 |
| S6 | 23 | 0.96 | 1.07 | 0 – 4 | 23 | 0.52 | 0.85 | 0 – 3 |
| S7 | 23 | 1.13 | 1.26 | 0 – 4 | 23 | 0.87 | 0.87 | 0 – 3 |
| How painful (P) |  |  |  |  |  |  |  |  |
| P1 | 23 | 2.78 | 1.09 | 0 – 4 | 23 | 0.15 | 1.08 | 0 – 4 |
| P2 | 23 | 2.00 | 1.13 | 0 – 4 | 23 | 0.70 | 0.88 | 0 – 3 |
| P3 | 23 | 1.43 | 1.24 | 0 – 4 | 23 | 0.30 | 0.77 | 0 – 3 |
| P4 | 23 | 1.57 | 1.41 | 0 – 4 | 23 | 0.74 | 1.01 | 0 – 3 |
| P6a | 23 | 2.04 | 1.40 | 0 – 4 | 23 | 0.96 | 0.98 | 0 – 3 |
| P6b | 23 | 1.83 | 1.37 | 0 – 4 | 23 | 0.61 | 0.99 | 0 – 3 |
| P8a | 23 | 1.87 | 1.42 | 0 – 4 | 23 | 0.52 | 0.90 | 0 – 3 |
| P9 | 23 | 1.78 | 1.38 | 0 – 4 | 23 | 0.78 | 1.09 | 0 – 4 |
| Difficulty during daily  activities (ADL) |  |  |  |  |  |  |  |  |
| A1 | 23 | 1.74 | 1.39 | 0 – 4 | 23 | 0.74 | 1.14 | 0 – 4 |
| A2 | 23 | 1.91 | 1.47 | 0 – 4 | 23 | 1.00 | 1.13 | 0 – 4 |
| A3 | 23 | 0.70 | 0.77 | 0 – 3 | 23 | 0.30 | 0.77 | 0 – 3 |
| A5 | 23 | 1.35 | 1.19 | 0 – 4 | 23 | 0.61 | 0.94 | 0 – 3 |
| A7 | 23 | 0.83 | 1.03 | 0 – 4 | 23 | 0.17 | 0.39 | 0 – 1 |
| A10 | 23 | 0.52 | 0.67 | 0 – 2 | 23 | 0.09 | 0.29 | 0 – 1 |
| A12 | 23 | 1.04 | 1.19 | 0 – 4 | 23 | 0.22 | 0.52 | 0 – 2 |
| A13 | 23 | 1.04 | 1.30 | 0 – 4 | 23 | 0.30 | 0.56 | 0 – 2 |
| A14 | 23 | 1.39 | 1.23 | 0 – 4 | 23 | 0.35 | 0.57 | 0 – 2 |
| A16 | 23 | 1.30 | 1.11 | 0 – 4 | 23 | 0.35 | 0.71 | 0 – 2 |
| A17 | 23 | 1.00 | 1.17 | 0 – 4 | 23 | 0.26 | 0.54 | 0 – 2 |
| Difficulty in sports and  playing (SP) |  |  |  |  |  |  |  |  |
| SP1 | 23 | 2.61 | 1.44 | 0 – 4 | 23 | 1.48 | 1.34 | 0 – 4 |
| SP2 | 23 | 3.09 | 1.17 | 0 – 4 | 23 | 1.48 | 1.47 | 0 – 4 |
| SP3 | 23 | 3.00 | 1.09 | 0 – 4 | 23 | 1.57 | 1.62 | 0 – 4 |
| SP4 | 23 | 2.26 | 1.29 | 0 – 4 | 23 | 0.96 | 1.19 | 0 – 4 |
| SP5 | 23 | 3.13 | 1.01 | 1 – 4 | 23 | 1.96 | 1.64 | 0 – 4 |
| SPN6 | 23 | 2.22 | 1.24 | 0 – 4 | 23 | 0.91 | 1.00 | 0 – 4 |
| SPN7 | 23 | 2.83 | 1.15 | 0 – 4 | 23 | 1.57 | 1.27 | 0 – 4 |
| Knee-related  quality of life (QoL) |  |  |  |  |  |  |  |  |
| Q1 | 23 | 3.04 | 0.56 | 1 – 4 | 23 | 1.61 | 0.89 | 0 – 3 |
| Q2 | 23 | 2.48 | 1.04 | 0 – 4 | 23 | 1.70 | 1.19 | 0 – 4 |
| Q3 | 23 | 2.30 | 0.93 | 1 – 4 | 23 | 1.30 | 0.77 | 0 – 3 |
| Q4 | 23 | 2.78 | 0.74 | 1 – 4 | 23 | 1.48 | 0.90 | 0 – 3 |
| QN5 | 23 | 2.13 | 1.01 | 0 – 4 | 23 | 0.87 | 1.06 | 0 – 3 |
| QN6 | 23 | 1.83 | 1.23 | 0 – 4 | 23 | 1.00 | 1.09 | 0 – 4 |

**Table C. Descriptive statistics for the KOOS-Child Score of the five subscales at T1 and T2 (Scale statistics)**

| Subscale | T1 | | | | T2 | | | |
| --- | --- | --- | --- | --- | --- | --- | --- | --- |
|  | *N* | *M* | *SD* | Range | *N* | *M* | *SD* | Range |
| S | 23 | 1.26 | 0.79 | 0.14 – 3.43 | 23 | 0.76 | 0.52 | 0.00 – 1.86 |
| P | 23 | 1.91 | 0.91 | 0.38 – 3.50 | 23 | 0.77 | 0.62 | 0.00 – 1.88 |
| ADL | 23 | 1.17 | 0.81 | 0.09 – 3.09 | 23 | 0.40 | 0.50 | 0.00 – 1.91 |
| SP | 23 | 2.73 | 1.95 | 0.29 – 4.00 | 23 | 1.42 | 0.74 | 0.00 – 3.71 |
| QoL | 23 | 2.43 | 0.73 | 1.00 – 3.67 | 23 | 1.33 | 0.74 | 0.00 – 3.33 |

S = knee problems; P = how painful; ADL = difficulty during daily activities; SP = difficulty in sports and playing; QoL = knee-related quality of life
